# Efectividad de la inmunización con nirsevimab en neonatos para prevenir hospitalizaciones por virus respiratorio sincitial durante dos temporadas en Navarra

**DOI:** 10.23938/ASSN.1133

**Published:** 2025-09-26

**Authors:** Noelia Vera-Punzano, Ana Navascués, Leticia Armendáriz, Natividad Viguria, Mercedes Herranz-Aguirre, Manuel García Cenoz, Camino Trobajo-Sanmartín, Aitziber Echeverria, Iván Martínez-Baz, Carmen Ezpeleta, Guillermo Ezpeleta, Jesús Castilla

**Affiliations:** 1 Instituto de Salud Pública y Laboral de Navarra Pamplona Navarra España; 2 Instituto de Investigación Sanitaria de Navarra (IdiSNA) Pamplona Navarra España; 3 CIBER de Epidemiología y Salud Pública España; 4 Servicio Navarro de Salud-Osasunbidea Hospital Universitario de Navarra Servicio de Microbiología Clínica Pamplona Navarra España; 5 Servicio Navarro de Salud-Osasunbidea Hospital Universitario de Navarra Servicio de Pediatría Pamplona Navarra España

**Keywords:** Virus Respiratorio Sincitial, Prevención, Nirsevimab, Hospitalización, Efectividad, Respiratory Syncytial Virus, Prevention, Nirsevimab, Hospitalisation, Effectiveness

## Abstract

**Fundamento:**

La infección por el virus respiratorio sincitial (VRS) es la principal causa de hospitalización en lactantes. En 2022 se autorizó el nirsevimab en la Unión Europea para prevenir la enfermedad respiratoria grave por VRS durante el primer año de vida. Evaluamos la efectividad de la inmunoprofilaxis con nirsevimab en recién nacidos para prevenir hospitalizaciones por VRS en Navarra durante las dos primeras temporadas de implementación.

**Métodos:**

Se ofreció nirsevimab a las cohortes de recién nacidos de octubre a diciembre de 2023 y de septiembre a diciembre de 2024, que se siguieron hasta febrero del año siguiente. Se consideraron casos a las hospitalizaciones por VRS confirmadas por PCR. Mediante regresión de Cox se estimó la razón de riesgos de hospitalización por VRS de inmunizados frente a no inmunizados.

**Resultados:**

Se ofreció nirsevimab a 2.699 recién nacidos, y 2.541 lo recibieron (94,1%). Se registraron 17 hospitalizaciones por VRS en la temporada 2023-2024 y 24 en la 2024-2025. El riesgo promedio de hospitalización por VRS fue 7,6% en no inmunizados y 1,1% en inmunizados. La efectividad promedio del nirsevimab para prevenir hospitalizaciones por VRS fue 79,5% (IC95%: 59,2-89,7), 89,9% en la temporada 2023-2024 y 52,8% en la 2024-2025, sin diferencias significativas entre ambas (p=0,055). La inmunoprofilaxis previno en promedio una hospitalización por VRS por cada 22,6 inmunizados.

**Conclusiones:**

La inmunoprofilaxis con nirsevimab resultó efectiva para prevenir hospitalizaciones por VRS y alivió la sobrecarga de ingresos pediátricos. Ante la posibilidad de casos en inmunizados, debe complementarse con otras medidas preventivas.

## Abstract (en)

**Background:**

Respiratory syncytial virus (RSV) is the leading cause of infant hospitalisation. In 2022, nirsevimab was approved in the European Union to prevent severe respiratory disease due to RSV the first year of life. Our aim is to evaluate the effectiveness of nirsevimab immunoprophylaxis in newborns for preventing RSV-related hospitalisations in Navarre, Spain, during its first two seasons of use.

**Methods:**

Nirsevimab was offered free of charge to infants born from October to December 2023 and from September to December 2024. Each cohort was followed until February of the following year. Cases were infants hospitalised for PCR-confirmed RSV infection. Cox regression was used to estimate the hazard ratio of hospitalisation for immunised versus non-immunised children.

**Results:**

Nirsevimab was offered to 2,699 newborns; of them, 2,541 (94.1%) received nirsevimab. In the 2023-2024 season, 17 RSV-related hospitalisations due to RSV were recorded and 24 in the 2024-2025 season. The average risk of hospitalisation was 7.6% in non-immunised newborns versus 1.1% in immunised ones. Overall, effec-tiveness of nirsevimab was 79.5% (95%CI: 59.2-89.7), with estimates of 89.9% in 2023-2024 and 52.8% in 2024-2025, with no significant diffe-rences between seasons (p=0.055). On average, one RSV hospitalisation was prevented per 22.6 immunised newborns.

**Conclusions:**

Nirsevimab immunoprophylaxis substantially reduces RSV hospitalisations, helping ease paediatric hospital burden. However, as some immunised infants were still hospitalised, additional preventive measures remain necessary.

## INTRODUCCIÓN

El virus respiratorio sincitial (VRS) es la causa más frecuente de infección respiratoria aguda en población pediátrica, especialmente en menores de seis meses^1^. El VRS causa cada año 33 millones de episodios respiratorios en todo el mundo en menores de cinco años, y 20 hospitalizaciones por 1.000 menores de seis meses^2^; en Navarra, dicha tasa se situó en 49 por 1.000 en la temporada 2022-2023^3^.

La infección por VRS es la principal causa de bronquiolitis en lactantes y puede ocasionar complicaciones a largo plazo, como asma o sibilancias recurrentes^1^^,^^4^. La circulación del VRS se concentra durante el invierno, generando una elevada carga de enfermedad en la infancia, con sobrecarga del sistema sanitario. En España, la mayor parte de las hospitalizaciones por VRS se producen entre noviembre y marzo, con la máxima incidencia en diciembre^5^. Sin embargo, tras la pandemia de COVID-19 se han observado cambios en los patrones típicos de circulación de los virus respiratorios, incluido el VRS^3^.

La Agencia Europea del Medicamento autorizó en octubre de 2022 el anticuerpo monoclonal nirsevimab (Beyfortus®) para la prevención de la enfermedad de vías respiratorias inferiores producida por VRS en neonatos y lactantes durante su primera temporada del VRS, y en niños menores de 24 meses con comorbilidades durante la segunda temporada^6^. Este anticuerpo monoclonal de vida media ampliada confiere protección durante al menos cinco meses tras la administración de una dosis, lo que supone una ventaja frente al palivizumab que requiere administración mensual^7^^,^^8^. En ensayos clínicos, el nirsevimab demostró una eficacia superior al 70% para prevenir casos confirmados de enfermedad por VRS^9^. La introducción del nirsevimab en la temporada 2023-2024 en algunos países, incluido España, demostró un buen perfil de seguridad tras la administración de más de 200.000 dosis^10^^-^^13^.

El objetivo de este estudio fue evaluar la efectividad y el impacto de la inmunoprofilaxis con nirsevimab en recién nacidos para prevenir la hospitalización por VRS en Navarra durante las temporadas 2023-2024 y 2024-2025.

## MATERIAL Y MÉTODOS

Se llevó a cabo un estudio de cohortes poblacionales prospectivas que incluyó a todos los recién nacidos en las temporadas 2023-2024 y 2024-2025 en Navarra, comunidad autónoma que cuenta alrededor de 680.000 habitantes y 4.800 nacimientos anuales.

El Comité de Ética de la Investigación con Medicamentos de Navarra aprobó el estudio (EO2023/12 y PI2024/150).

La inmunoprofilaxis con nirsevimab se ofreció gratuitamente en los servicios materno-infantiles de los hospitales a todos los recién nacidos entre el 1 de octubre de 2023 y el 5 de febrero de 2024^14^, y entre el 1 de septiembre de 2024 y el 16 de febrero de 2025. También se ofreció nirsevimab a aquellos residentes en Navarra nacidos fuera de la región durante los periodos mencionados. Se administró una dosis de 50 mg por vía intramuscular a neonatos con menos de 5 kg de peso y una dosis de 100 mg a aquellos con 5 kg o más. Todos los recién nacidos inmunizados y los no inmunizados por negativa de progenitores o tutores se incluyeron en el Registro de Vacunaciones. La inmunización se consideró potencialmente efectiva un día después de su administración.

Previo al ingreso hospitalario por infección respiratoria aguda, se realizó sistemáticamente una determinación del VRS por reacción en cadena de la polimerasa (PCR). Los resultados se notificaron electrónicamente al sistema de vigilancia epidemiológica reforzada. Todos los casos de lactantes hospitalizados más de 24 horas con infección por VRS confirmada por PCR fueron revisados por médicos de salud pública, y solo se tuvieron en cuenta los ingresos debidos a infección del tracto respiratorio inferior por VRS. Entre los hospitalizados por VRS se distinguieron aquellos que requirieron ingreso en unidades de cuidados intensivos (UCI) pediátricas.

La relación de recién nacidos se obtuvo de bases de datos administrativas y se relacionó con la de las hospitalizaciones por VRS y la de las profilaxis con nirsevimab mediante el número de identificación del paciente. El análisis estadístico se realizó con datos anonimizados.

### Análisis estadístico

El periodo de estudio comprendió dos cohortes prospectivas de nacidos del 1 de octubre al 31 de diciembre de 2023 y del 1 de septiembre al 31 de diciembre de 2024. No se incluyeron nacidos a partir de enero por no haberse registrado ningún ingreso hospitalario por VRS entre ellos^14^^,^^15^. El seguimiento comenzó en la fecha de nacimiento y finalizó en la fecha de la prueba que dio lugar al diagnóstico del caso, y para el resto de los participantes, el último día de la semana en la que se registró el último caso hospitalizado de la temporada. Esta fecha fue el 18 de febrero de 2024 para los nacidos en la temporada 2023-2024, y el 26 de enero de 2025 para los nacidos en la temporada 2024-2025.

Se calculó el riesgo y la tasa de incidencia de hospitalización y de ingreso en UCI pediátrica según el antecedente de inmunización con nirsevimab para el promedio de ambas temporadas, para cada sexo y cada temporada. El número de nacimientos registrados en Navarra se utilizó como denominador de las tasas de hospitalización. El riesgo en inmunizados y no inmunizados se comparó mediante la prueba de Chi-cuadrado o el test exacto de Fisher.

Para estimar la efectividad del nirsevimab para prevenir hospitalizaciones por VRS, se comparó la tasa de hospitalización por infección confirmada por VRS en los inmunizados y no inmunizados con nirsevimab mediante modelos de regresión de Cox y se obtuvo la razón de riesgos (*hazard ratio*, HR) con su intervalo de confianza del 95% (IC95%). Se utilizó el número de días contados a partir del 1 de octubre de 2023 o del 1 de septiembre de 2024, según la temporada, como escala temporal subyacente de los modelos. El estado de inmunización con nirsevimab se definió como una variable cambiante en el tiempo, considerando no inmunizados a los recién nacidos hasta el día anterior a la administración e inmunizados desde un día después de la administración. Las personas-día a riesgo se utilizaron como denominador de las tasas de incidencia. Los modelos se ajustaron por sexo y semana de nacimiento. La efectividad se estimó como (1 - HR) × 100. De igual forma, se evaluó la efectividad del nirsevimab para prevenir ingresos en la UCI pediátrica a causa de la infección por VRS. Estos análisis se llevaron a cabo para cada temporada por separado y para el conjunto de ambas.

El número de hospitalizaciones por VRS registradas y la estimación promedio de la efectividad del nirsevimab se utilizaron para estimar el número de hospitalizaciones que se hubiera producido en ausencia de inmunoprofilaxis en cada temporada, dividiendo el número de hospitalizaciones en inmunizados entre el HR de hospitalización por el VRS. El número estimado de hospitalizaciones prevenidas por el nirsevimab se calculó restando el número observado al número estimado en ausencia de inmunoprofilaxis. El número de recién nacidos que tuvieron que ser inmunizados con nirsevimab para prevenir una hospitalización por VRS se calculó como el cociente entre el número de inmunoprofilaxis administradas y el número estimado de hospitalizaciones prevenidas. Para estimar el coste económico en dosis de nirsevimab para prevenir una hospitalización se consideró el precio de 209 euros por dosis.

## RESULTADOS

### Características de las cohortes de recién nacidos

El estudio incluyó 2.699 recién nacidos, 1.183 en la temporada 2023-2024 y 1.516 en la 2024-2025. Se inmunizó con nirsevimab al 94,1% de recién nacidos, con una cobertura algo mayor en la temporada 2024-2025 que en la 2023-2024 (95,8% vs. 92,1%, p<0,001). La población de estudio incluyó más niños que niñas (53,1% vs. 46,9%), pero sin diferencias significativas en la cobertura de inmunización por sexo (p=0,855).

En la temporada 2023-2024 se registraron 17 hospitalizaciones por VRS, distribuidas entre el 11 de noviembre de 2023 y el 13 de febrero de 2024. En la temporada 2024-2025 se registraron 24 hospitalizaciones por VRS, que se distribuyeron desde el 20 de octubre de 2024 hasta el 24 de enero de 2025 (Tabla 1).

**Tabla 1:** Riesgo y tasa de hospitalización y de ingreso en unidad de cuidados intensivos pediátrica por infección confirmada por virus respiratorio sincitial en neonatos inmunizados y en no inmunizados con nirsevimab en las temporadas 2023-2024 y 2024-2025 en Navarra

	Nacidos n	Personas-día	Casos n	Riesgo %	Tasa*
Hospitalización					
Ambas temporadas					
No inmunizados	158	21.161	12	7,59	0,57
Inmunizados	2.541	223.266	29	1,14	0,13
Hombres					
No inmunizados	85	11.412		9,41	0,7
Inmunizados	1.348	118.226	13	0,96	0,11
Mujeres					
No inmunizados	73	9.748		5,48	0,41
Inmunizados	1.193	105.041	16	1,34	0,15
Temporada 2023-2024					
No inmunizados	94	11.248		9,57	0,8
Inmunizados	1.089	100.614		0,73	0,08
Temporada 2024-2025					
No inmunizados	64	9.913	3	4,69	0,3
Inmunizados	1.452	122.652	21	1,45	0,17
Ingreso en UCI pediátrica					
Ambas temporadas					
No inmunizados	158	21.161	4	2,53	0,19
Inmunizados	2.541	223.266	16	0,63	0,07
Temporada 2023-2024					
No inmunizados	94	11.248	2	2,13	0,18
Inmunizados	1.089	100.614	3	0,28	0,03
Temporada 2024-2025					
No inmunizados	64	9.913	2	3,13	0,2
Inmunizados	1.452	122.652	13	0,9	0,11

*: tasa por 1.000 personas-día; UCI: unidad de cuidados intensivos.

### Efectividad del nirsevimab para prevenir hospitalizaciones por VRS

Entre las dos cohortes estudiadas hubo 41 ingresos hospitalarios por VRS, de los cuales 20 fueron ingresados en la UCI pediátrica. De los casos hospitalizados, 29 estaban inmunizados con nirsevimab (70,7%), 8 en la temporada 2023-2024 y 21 en la 2024-2025. El riesgo de hospitalización por VRS para el promedio de las dos temporadas fue del 7,6% entre los no inmunizados y 1,1% entre los inmunizados; los riesgos de ingreso en UCI pediátrica fueron del 2,5% y 0,6%, respectivamente (Tabla 1).

**Figura 1:**
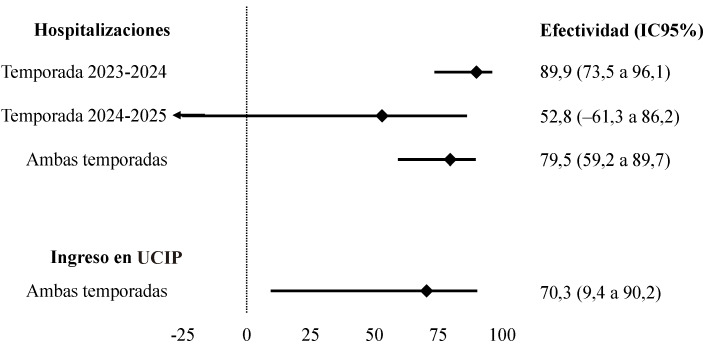
Efectividad del nirsevimab para prevenir hospitalizaciones e ingresos en la unidad de cuidados intensivos pediátrica debidos a infección por virus respiratorio sincitial durante las temporadas 2023-2024 y 2024-2025 en Navarra.

En el análisis multivariante, la estimación promedio de efectividad del nirsevimab para la prevención de hospitalizaciones por VRS fue del 79,5% (IC95%: 59,2 a 89,7). La efectividad en la temporada 2023-2024 fue de 89,9% (IC95%: 73,5 a 96,1) y en la temporada 2024-2025 del 52,8% (IC95%: -61,3 a 86,2), sin alcanzarse diferencias estadísticamente significativas entre ambas (p=0,055). Para el conjunto de las dos temporadas, las estimaciones de efectividad fueron del 65,4% (IC95%: -7,0 a 88,8) en mujeres y del 86,2% (IC95%: 65,4 a 94,5) en hombres. La efectividad promedio para prevenir ingresos en UCI pediátrica por VRS fue del 70,3% (IC95%: 9,4 a 90,2) (Tabla 2 y Fig. 1).

**Tabla 2:** Efecto de la inmunización con nirsevimab sobre el riesgo de hospitalización y de ingreso en la unidad de cuidados intensivos pediátrica por infección debida a virus respiratorio sincitial en las temporadas 2023-2024 y 2024-2025 en Navarra

	HR (IC 95%)	
	Inmunizados frente a no inmunizados	
Población analizada	Crudo	Ajustado*
*Hospitalización*		
Total	0,230 (0,117-0,453)	0,205 (0,103-0,408)
Hombres	0,155 (0,064-0,375)	0,138 (0,055-0,346)
Mujeres	0,383 (0,127-1,151)	0,346 (0,112-1,070)
Temporada 2023-2024	0,107 (0,041-0,278)	0,101 (0,039-0,265)
Temporada 2024-2025	0,555 (0,165-1,868)	0,472 (0,138-1,613)
*Ingreso en UCI pediátrica*		
Total	0,387 (0,129-1,166)	0,297 (0,098-0,906)

HR: *hazard ratio* (razón de riesgos); IC95%: intervalo de confianza del 95%; *: por sexo y semana de nacimiento; UCI: unidad de cuidados intensivos.

IC95%: intervalo de confianza del 95%; UCIP: unidad de cuidados intensivos pediátrica.

Las 41 hospitalizaciones debidas al VRS registradas entre las dos temporadas supusieron una tasa de 15,2 por 1.000 nacidos, y los 20 ingresados en la UCI pediátrica una tasa de 7,4 por 1.000 nacidos. La inmunoprofilaxis con nirsevimab evitó un número estimado de 112,5 (73,3%) hospitalizaciones (41,7 por 1.000 nacidos) y 37,9 (65,4%) ingresos en UCI pediátrica (14,0 por 1.000 nacidos). Estos resultados permiten estimar que se evitó una hospitalización por VRS por cada 22,6 recién nacidos inmunizados con nirsevimab y un ingreso en UCI pediátrica por cada 67,1 inmunizados (Tabla 3). El coste en dosis de nirsevimab fue de 4.722 euros para prevenir un ingreso hospitalario por VRS y de 14.023 euros para prevenir un ingreso en UCI pediátrica.

**Tabla 3:** Impacto estimado de la inmunoprofilaxis con nirsevimab en las cohortes de nacidos en las temporadas 2023-2024 y 2024-2025 en Navarra

	Hospitalización			UCIP
Temporada	2023-2024	2024-2025	Ambas	Ambas
*Eventos observados*				
n (%)	17 (35,4)	24 (22,8)	41 (26,7)	20 (34,6)
Tasa por 1.000 nacidos	14,4	15,8	15,2	7,4
Eventos prevenidos*					
n (%)	31,0 (64,6)	81,4 (77,2)	112,5 (73,3)	37,9 (65,4)
Tasa por 1.000 nacidos	26,2	53,7	41,7	14
Eventos totales en ausencia de inmunoprofilaxis*				
n	48	105,4	153,5	57,9
Tasa por 1.000 nacidos	40,6	69,6	56,9	21,4
Inmunizados con nirsevimab para prevenir un caso**				
n	35,1	17,8	22,6	67,1

UCIP: unidad de cuidados intensivos pediátrica; *: el número de hospitalizaciones que se hubiera producido en ausencia de inmunoprofilaxis se estimó dividiendo el número de hospitalizaciones en inmunizados entre el *hazard ratio* de hospitalización por virus respiratorio sincitial promedio de las dos temporadas. El número estimado de hospitalizaciones prevenidas por el nirsevimab se calculó restando el número observado al número estimado en ausencia de inmunoprofilaxis; **: el número de recién nacidos que tuvieron que ser inmunizados con nirsevimab para prevenir una hospitalización por virus respiratorio sincitial se calculó como el cociente entre el número de inmunoprofilaxis administradas y el número estimado de hospitalizaciones prevenidas.

## DISCUSIÓN

En el promedio de dos temporadas, la inmunoprofilaxis con nirsevimab en recién nacidos fue altamente efectiva (79,5%) para prevenir la hospitalización por infección del tracto respiratorio inferior por VRS. La efectividad también fue elevada para la prevención de casos que requieren ingreso en UCI pediátrica (70,3%).

En el ensayo clínico MELODY, el nirsevimab demostró una eficacia del 76,8% para la prevención de hospitalizaciones por infección del tracto respiratorio inferior asociada a VRS^9^. En el ensayo HARMONIE, un estudio de eficacia de fase 3b en condiciones reales, dicha eficacia fue del 83,2%^16^. Las estimaciones de efectividad obtenidas para el promedio de las dos primeras temporadas en Navarra fueron consistentes con las obtenidas en estos ensayos clínicos.

Nuestras estimaciones están dentro del rango de los resultados obtenidos en condiciones de uso real en la temporada 2023-2024 en España^10^^,^^17^^-^^20^, en otros países europeos^11^^,^^13^^,^^21^^,^^22^ y en Estados Unidos^23^^,^^24^. Estos estudios encontraron estimaciones de efectividad de entre el 55% y el 90% para la prevención de hospitalizaciones asociadas al VRS en los lactantes. Hasta donde conocemos, aún no se han publicado resultados de efectividad de la temporada 2024-2025.

La cobertura promedio de inmunización con nirsevimab en Navarra fue muy alta (94%), dentro del rango de las coberturas alcanzadas en otras comunidades autónomas en la temporada 2023-2024, que oscilaron entre el 79% y el 99%^10^^,^^17^^-^^20^. Esta buena cobertura pudo deberse a la estrategia de inmunización a los recién nacidos en el paritorio o en los primeros días de vida, antes del alta de la planta de maternidad. De este modo, se aseguró el contacto e información temprana a las familias por parte de un profesional sanitario y se redujo el número de puntos de administración. Núñez y col encontraron mayor cobertura de inmunización de recién nacidos en las unidades materno-infantiles de los hospitales que en atención primaria^25^. La financiación pública y gratuita para las familias es otro factor que puede haber contribuido a las altas coberturas.

La temporada 2023-2024 de VRS en Navarra tuvo una presentación ligeramente adelantada con respecto a lo que era habitual antes de la pandemia de COVID-19^5^, y la incidencia de VRS fue entre moderada y baja. La inmunoprofilaxis con nirsevimab se indicó prospectivamente a los nacidos a partir de octubre. La estimación de la efectividad fue algo alta en comparación con los resultados de los ensayos clínicos, aunque los amplios intervalos de confianza no permitieron concluir que los resultados fueran realmente mejores^12^.

La temporada 2024-2025 fue todavía más temprana en presentación y la incidencia de VRS fue más alta en todas las edades^15^. La indicación de la inmunoprofilaxis se adelantó para incluir a los nacidos desde septiembre. Sin que se hayan detectado diferencias estadísticamente significativas, la estimación de la efectividad del nirsevimab para prevenir hospitalizaciones por VRS en esta segunda temporada fue moderada y no alcanzó significación estadística. La estimación conjunta de ambas temporadas se situó en valores similares a los de los ensayos clínicos y estudios publicados. Ante la ausencia de explicaciones biológicas o médicas, la aparente discrepancia en la efectividad entre las dos temporadas en Navarra que no llega a ser estadísticamente significativa podría explicarse por la variabilidad aleatoria de los resultados obtenidos en estudios con un número de sujetos no muy grande.

Se detectaron hospitalizaciones por VRS en veintinueve lactantes que habían recibido la inmunoprofilaxis. Esto pone de manifiesto la necesidad de complementar esta intervención con otras medidas preventivas en torno a los lactantes, como reducir el número de contactos con otras personas durante las épocas de circulación del VRS, evitar la proximidad de personas que tengan síntomas respiratorios, y reforzar la higiene de manos y de objetos, así como el uso de mascarillas^26^^,^^27^.

Destaca el importante impacto de esta intervención, que evitó el 73% de las hospitalizaciones y el 65% de los ingresos en UCI pediátrica por VRS entre las dos temporadas. Su prevención contribuyó a aliviar apreciablemente la asistencia hospitalaria pediátrica en semanas en las que habría habido una especial sobrecarga.

Demostrada la alta efectividad del nirsevimab, la principal limitación para su extensión es el elevado precio por dosis, teniendo en cuenta que la mayoría de los recién nacidos a los que se administra no van a desarrollar enfermedad grave por el VRS^28^. No obstante, la estrategia evaluada consiguió prevenir un ingreso hospitalario a un coste de 4.722 euros y un ingreso en UCI pediátrica a un coste de 14.023 euros, demostrándose como una intervención muy eficiente. Estos resultados apoyan el mantener esta intervención en las próximas temporadas, y abren el camino a valorar la posible ampliación de la indicación y financiación de la inmunoprofilaxis a los nacidos en otros meses, ya que podría ser una estrategia coste-efectiva para el sistema sanitario.

Este estudio tiene varias fortalezas, como el reclutamiento prospectivo de recién nacidos y su seguimiento durante semanas en periodos de alta circulación del VRS. El estudio incluyó a toda la población diana de la intervención, lo que evita posibles sesgos y garantiza una buena representatividad. La captación de casos se basó en la confirmación sistemática mediante PCR de los casos sospechosos de infección por VRS que iban a ingresar en el hospital. Los casos fueron obtenidos de la vigilancia epidemiológica reforzada de esta infección, que se basa en una notificación automática de los casos confirmados en los hospitales. El análisis de dos temporadas consecutivas de VRS proporciona resultados promedio, aumenta la precisión de las estimaciones y disminuye la influencia de particularidades de cada temporada. El análisis de regresión de Cox tomando como variable de tiempo la fecha de calendario compara la incidencia de inmunizados con la de no inmunizados en el mismo momento, lo que controla el sesgo que podría introducir la variabilidad en la incidencia de VRS a lo largo del tiempo.

Entre las limitaciones de este estudio se encuentra la circunstancia de que el bajo número de recién nacidos cuyos padres rechazaron la inmunización limitó la potencia estadística del estudio; sin embargo, el análisis conjunto de dos temporadas consiguió un tamaño suficiente para obtener estimaciones precisas. El tamaño de los subanálisis, como el de cada temporada por separado, puede tener potencia estadística insuficiente, por lo que los resultados han de interpretarse con cautela. La decisión de ingreso hospitalario puede variar en función del profesional sanitario, del centro y de las camas disponibles en cada momento, lo que podría haber influido en las estimaciones de efectividad. Las comorbilidades y la prematuridad podrían ser factores de confusión en el análisis, pero estas variables no estuvieron disponibles para todos los recién nacidos y no se incluyeron en los análisis. En Navarra, el nirsevimab también se ofreció a todos los niños menores de 24 meses con comorbilidades; sin embargo, no se incluyeron en este análisis ni se solaparon con los lactantes incluidos en este estudio. Aunque este estudio se ha llevado a cabo en una región, las estimaciones de efectividad del nirsevimab para prevenir la hospitalización pueden ser aplicables a otros lugares. No ocurre lo mismo con las estimaciones de eventos prevenidos y número de inmunizaciones necesarias, que están condicionadas por las características y epidemiología del lugar. El elevado coste del nirsevimab hace necesaria una evaluación económica que determine la estrategia óptima de uso específica de cada país.

En conclusión, la inmunoprofilaxis con nirsevimab de los nacidos entre septiembre y diciembre fue muy efectiva para prevenir hospitalizaciones e ingresos en UCI pediátrica por VRS, disminuyendo la sobrecarga de actividad hospitalaria pediátrica en los meses de mayor circulación de virus respiratorios. Los resultados muestran una alta eficiencia de esta intervención para prevenir hospitalizaciones por VRS. El riesgo de enfermar no desaparece en inmunizados, lo que hace recomendable complementar la inmunización con otras medidas preventivas.

## Data Availability

El acceso a los datos de este estudio requiere autorización del Departamento de Salud del Gobierno de Navarra y aprobación por el Comité de Ética de la Investigación con Medicamentos de Navarra.
